# Association of *TLR* variants with susceptibility to *Plasmodium vivax* malaria and parasitemia in the Amazon region of Brazil

**DOI:** 10.1371/journal.pone.0183840

**Published:** 2017-08-29

**Authors:** Allyson Guimarães Costa, Rajendranath Ramasawmy, Hiochelson Najibe Santos Ibiapina, Vanderson Souza Sampaio, Lilyane Amorim Xábregas, Larissa Wanderley Brasil, Andréa Monteiro Tarragô, Anne Cristine Gomes Almeida, Andrea Kuehn, Sheila Vitor-Silva, Gisely Cardoso Melo, André Machado Siqueira, Wuelton Marcelo Monteiro, Marcus Vinicius Guimarães Lacerda, Adriana Malheiro

**Affiliations:** 1 Programa de Pós-Graduação em Medicina Tropical, Universidade do Estado do Amazonas (UEA), Manaus, AM, Brazil; 2 Diretoria de Ensino e Pesquisa, Fundação de Medicina Tropical Dr. Heitor Vieira Dourado (FMT-HVD), Manaus, AM, Brazil; 3 Laboratório de Genômica, Fundação Hospitalar de Hematologia e Hemoterapia do Amazonas (HEMOAM), Manaus, AM, Brazil; 4 Programa de Pós-Graduação em Imunologia Básica e Aplicada, Universidade Federal do Amazonas (UFAM), Manaus, AM, Brazil; 5 Universidade Nilton Lins (UNINILTONLINS), Manaus, AM, Brasil; 6 Barcelona Centre for International Health Research (CRESIB), Barcelona Global Health Institute (ISGLOBAL), Barcelona, Spain; 7 Instituto Nacional de Infectologia Evandro Chagas, Fundação Oswaldo Cruz (FIOCRUZ), Rio de Janeiro, RJ, Brazil; 8 Instituto de Pesquisas Leônidas & Maria Deane, FIOCRUZ-Amazônia, Manaus, AM, Brazil; Centro de Pesquisas Rene Rachou, BRAZIL

## Abstract

**Background:**

*Plasmodium vivax* malaria (*Pv*-malaria) is still considered a neglected disease despite an alarming number of individuals being infected annually. Malaria pathogenesis occurs with the onset of the vector-parasite-host interaction through the binding of pathogen-associated molecular patterns (PAMPs) and receptors of innate immunity, such as toll-like receptors (TLRs). The triggering of the signaling cascade produces an elevated inflammatory response. Genetic polymorphisms in TLRs are involved in susceptibility or resistance to infection, and the identification of genes involved with *Pv*-malaria response is important to elucidate the pathogenesis of the disease and may contribute to the formulation of control and elimination tools.

**Methodology/Principal findings:**

A retrospective case-control study was conducted in an intense transmission area of *Pv*-malaria in the state of Amazonas, Brazil. Genetic polymorphisms (SNPs) in different *TLRs*, *TIRAP*, and *CD14* were genotyped by polymerase chain reaction-restriction fragment length polymorphism (PCR-RFLP) analysis in 325 patients infected with *P*. *vivax* and 274 healthy individuals without malaria history in the prior 12 months from the same endemic area. Parasite load was determined by qPCR. Simple and multiple logistic/linear regressions were performed to investigate association between the polymorphisms and the occurrence of *Pv*-malaria and parasitemia. The C/T (*TLR5 R392StopCodon*) and T/T (*TLR9 -1486C/T*) genotypes appear to be risk factors for infection by *P*. *vivax* (*TLR5*: C/C vs. C/T [OR: 2.116, 95% CI: 1.054–4.452, *p* = 0.031]; *TLR9*: C/C vs. T/T [OR: 1.919, 95% CI: 1.159–3.177, *p* = 0.010]; respectively). Fever (COEF = 7599.46, 95% CI = 3063.80–12135.12, *p* = 0.001) and the C/C genotype of *TLR9 -1237C/T* (COEF = 17006.63, 95% CI = 3472.83–30540.44, *p* = 0.014) were independently associated with increased parasitemia in patients with *Pv*-malaria.

**Conclusions:**

Variants of TLRs may predispose individuals to infection by *P*. *vivax*. The *TLR5 R392StopCodon* and *TLR9 -1486C/T* variants are associated with susceptibility to *Pv-*malaria. Furthermore, the *TLR9* variant *-1237C/C* correlates with high parasitemia.

## Introduction

Approximately 214 million cases of malaria were diagnosed in 2015, with 438,000 deaths [1]. In Brazil, 140,000 cases were reported, representing 41.7% of cases in the Americas [2,3]. The Amazon region contributes nearly 99.9% of the malaria notifications, and *Plasmodium vivax* is responsible for 83.7% of cases [4,5].

Malaria results in a wide spectrum of clinical manifestations that occur during the vector-parasite-host interaction, and the first asexual reproduction process occurs in the liver [6,7]. Febrile episodes of malaria start after the interaction between the toxins that are produced by the schizont and released during the rupture of red blood cells and the phagocytic cells of the innate immune system [8]. These toxins, also called pathogen associated molecular patterns (PAMPs) are mainly recognized by toll-like receptors (TLRs) [9]. The *Plasmodium* PAMPs such as the glycosylphosphatidylinositol anchors (GPI), hemozoin linked to DNA are recognized by TLR-1/TLR-2, TLR-4, TLR-2/TLR-6, and TLR-9, respectively, producing an intense inflammatory response and activating dendritic cells, monocyte subtypes, and macrophages [10–16].

The inflammatory response resulting from the pathogenesis of the disease is closely related to the parasite load and the genetic background of the host [17]. Inflammation and cytokine production were reported to be higher in *P*. *vivax* infection than that in other species such as *Plasmodium falciparum* [18–20]. Deletion of TLRs and co-stimulatory molecules in murine models showed decreased production of proinflammatory cytokines and increased susceptibility to infection by different species of *Plasmodium* and other protozoans [11,21–23].

Genetic polymorphisms in TLRs are involved in cytokine activation pathways and may play a role in resistance or susceptibility to *Plasmodium* infection. The variant *TLR1 I602S* was associated with the development of symptomatic malaria and high parasitemia in *P*. *falciparum* malaria (*Pf*-malaria) [24,25]. Single nucleotide polymorphisms (SNPs) in *TLR4* (*A299G* and *T399I*) were shown to be associated with the onset of clinical manifestations of severe *Pf*-malaria in African children and pregnant women, and in adults with non-severe symptomatic malaria [26–28]. Individuals with the SNP *R392StopCodon* (*TLR5*) are unable to induce the intracellular signaling cascade in bacterial diseases, producing lower concentrations of inflammatory cytokines such as IL-6 and TNF-α [29,30]. Cytokines are important for parasite load control and *Plasmodium* clearance in humans [15,31]

The *TLR6 S249P* polymorphism may be a risk factor for the development of malaria [24]. Allelic variants in the promoter region of *TLR9 -1237C/T* and *-1486C/T* are associated with parasitemia in *Pf*-infected individuals and placental malaria [24,27]. The SNP of TIR domain-containing adaptor protein (*TIRAP*) *S180L* appears to confer protection against malaria, tuberculosis, and bacterial diseases [32]. CD14–*159* is associated with the incidence and mortality of septic shock [33,34].

*P*. *vivax* has several PAMPs that are recognized by TLRs, and studies have shown that polymorphisms in the *TLR* genes may be associated with *Pf*-malaria. In this study, SNPs in the *TLRs*, *TIRAP*, and *CD14* genes were investigated in *P*. *vivax* infected individuals from the Amazon region of Brazil. Association of the polymorphisms *TLR5 R392StopCodon* and *TLR9 -1486C/T* with *Pv-*malaria and the C/C genotype of the SNP *TLR9 -1237C/T* with increased parasitemia was observed.

## Materials and methods

### Study area and sampling

The study was conducted with biological samples collected from individuals of two areas of the state of Amazonas, based on the low intra-regional migration of its inhabitants and similar profiles of malaria transmission. The regions chosen were followed in two cohort studies, entitled "The epidemiology of malaria in the municipality of Careiro", and "The comparative epidemiology of *P*. *falciparum* and *P*. *vivax* transmission in Papua New Guinea, Thailand and Brazil", carried out in rural and peri-urban areas of the cities of Careiro and Manaus from 2008 to 2011 and 2013 to 2014, respectively.

The Careiro municipality, which is 110 km from the Amazonas state capital Manaus, has access to a federal highway (BR-319), and has an estimated population of 30,000. Most inhabitants live in rural areas and are supported by federal programs that encourage the practice of agriculture. The Panelão and Sítio Castanho communities comprise an estimated population of 1,200, and were chosen for the study due to the observation of intense transmission of *Pv-*malaria [35].

The communities Brasileirinho, Ipiranga, and Puraquequara, located in the peri-urban area east of Manaus, had an estimated population of 2,500 at the end of 2012, according to a census conducted by the FMT-HVD team shortly before our sample collection. There is uncontrolled deforestation in these areas. The economy is mainly based on agricultural and extraction activities, and is at high risk of *Pv*-infection [36].

**Fig 1:**
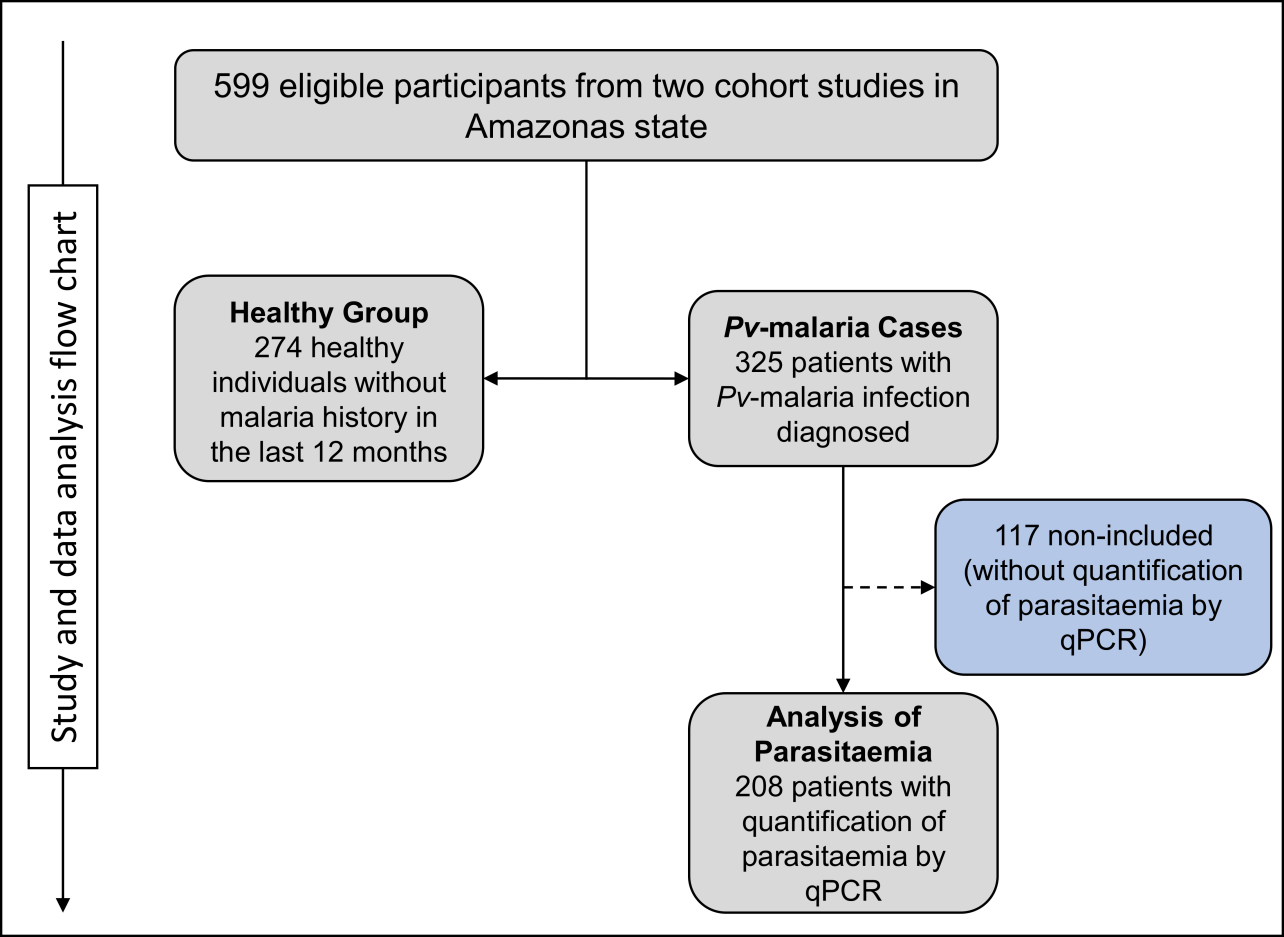
Study and data analysis flow chart. We included 599 eligible participants from two cohort studies conducted in Amazonas. Of these, 274 healthy individuals without malaria history in the prior 12 months were included in the “Healthy Group” and 325 patients diagnosed with *Pv*-malaria infection were included in the “*Pv*-malaria Cases”. Parasitemia analysis was performed for 208 patients.

A retrospective case-control study was conducted from the two cohorts. A total of 325 patients with *Pv*-malaria diagnosed by thick blood smear examination [37] and confirmed by qPCR [38] were included in the study, along with 274 healthy individuals with no malaria history in the prior 12 months and confirmed negative by qPCR for *Plasmodium* spp. during the cohort studies (Fig 1). All study participants were from the same endemic area, sharing similar environments and risk of exposure to the parasite.

### Ethics statement

The studies were approved by the Comitê de Ética em Pesquisa da Fundação de Medicina Tropical Dr. Heitor Vieira Dourado (CEP/FMT-HVD process #51536/2012), and by the Comissão Nacional de Ética em Pesquisa (CONEP) linked to the Conselho Nacional de Saúde (CONEP process #15197/2008, #349211/2013). All participants read and signed the written informed consent form. Malaria cases detected in longitudinal studies were treated in accordance with the recommendations of the Brazilian Ministry of Health [39].

### Genomic DNA extraction

Samples of 300 μL of blood were collected via finger puncture from each participant for genomic DNA purification. The QIAmp DNA kit (QIAGEN, Chatsworth, CA, USA) was used for the Careiro study, and the FavorPrep^TM^ 96-well Genomic DNA Kit (Favorgen, Ping-Tung, Taiwan) was used for the Manaus study. DNA samples were quantified with a NanoDrop 2000c (Thermo Fisher Scientific, Waltham, MA, USA) to evaluate the concentration, and purity of nucleic acids.

### Quantification of *P*. *vivax* DNA by qPCR assay

Parasitemia of *Pv*-malaria was determined by amplifying the 18S rRNA gene using the 7500 Fast qPCR System (Applied Biosystems, Foster, CA, USA) as described previously [40,41], and is expressed as number of copies/μL. The primers/probes, qPCR cycling conditions, qPCR efficiency and detection limit are shown in S1 Table. Parasitemia was obtained for only 208 *Pv*-malaria patients (Fig 1).

### Polymorphism genotyping

The following polymorphisms, *TLR1 I602S*, (rs5743618), *TLR4 A299G* (rs4986790), *TLR4 T399I* (rs4986791), *TLR5 R392StopCodon* (rs5744105), *TLR6 S249P* (rs5743810), *TLR9 -1237C/T* (rs187084), *TLR9 -1486C/T* (rs5743836), *TIRAP S180L* (rs8177374), and *CD14*–*159* (rs2569191) were investigated. Polymerase chain reaction-restriction fragment length polymorphism (PCR-RFLP) analysis was used for allelic discrimination as described previously [24,42,43]. Briefly, the PCR reaction for each SNP consisted of 1 μL genomic DNA (~ 20ng) added to 24 μL amplification mix containing 0.2 μL (2 U) Platinum™ Taq polymerase (Thermo Fisher Scientific), 2.5 μL 10x buffer (100 mmol/L Tris-HCl [pH 8.3] and 500 mmol/L KCl), 1 μL MgCl_2_ (1.5 mmol/L), 1 μL dNTPs (40 mmol/L), 0.5 μL each of forward and reverse primer (0.25 pmol/L) and 18.3 μL ultrapure dH_2_O. A total of 10 μL of PCR product was digested with 5 U of respective restriction endonuclease (New England Biolabs, Ipswich, MA, USA) in enzyme buffer according to the manufacturer’s instructions. The primers, PCR cycling conditions, and restriction endonucleases are shown in S2 Table. The fragments generated by PCR-RFLP were separated by electrophoresis in either a 2% or 4% agarose gel stained with GelRed™ Nucleic Acid Gel Stain (Biotium, Hayward, CA, USA), and visualized with the UV light Gel Doc™ XR+ System (Bio-Rad Corporation, Hercules, CA, USA) with a photo documentation system.

### Statistical and data analysis

Comparison between groups was performed with the chi-squared (χ^2^) or Fisher’s exact test with 95% confidence interval [CI]. The Hardy-Weinberg equilibrium (HWE) was determined by comparing the frequency of the observed and expected number of genotypes. Simple and multiple logistic regressions were performed to investigate association between the polymorphisms and the occurrence and recurrence of *Pv-*malaria and parasitemia. For both regression analyses, the variables age, gender, fever, and hemoglobin levels were included as confounders. A backward stepwise technique was applied. Variables with p-values less than or equal to 0.2 in the simple linear regression were selected for the multivariate model analysis. The final model considered all variables that were statistically significant (*p*<0.05). The analysis of haplotypes and linkage disequilibrium (LD) was carried out by Haploview software (v4.2). Tests for Hardy-Weinberg equilibrium were performed by an online application (https://ihg.gsf.de/cgi-bin/hw/hwa1.pl). Regression models were performed by Stata software (v13).

## Results

### Baseline characteristics of the study population

Baseline characteristics of the study population are shown in Table 1. The median age of the healthy control and *Pv*-malaria cases were 39 and 37 years, respectively (*p* = 0.744). In both groups, male subjects were predominant (55% and 62%). The average parasite load was 188 copies/uL. A median of 2 malarial episodes was reported by the patients. Approximately 27% of the *Pv*-malaria cases were primary infections. Patients reported fever (54%), headache (31%), chills (29%), myalgia (28%) and sweating (16%). The median hemoglobin level was 13.7 g/dL.

**Table 1:** Clinical and demographic characteristics of the study population.

Variables	Healthy Group	*Pv*-malaria Cases
	(n = 274)	(n = 325)
**Age** (years, median [IQR])	39 [20–57]	37 [19–53]
**Gender** (male/female)	151/123	200/125
**Parasitemia** (number of copies/μL, median [IQR])	-	188 [25.5–2,881.5]
**Number of infections** (median [IQR])	-	2 [0–8]
**First infection** (yes/no)	-	89/236
**Fever** (yes/no)	-	177/148
**Headache** (yes/no)	-	102/223
**Chills** (yes/no)	-	95/230
**Myalgia** (yes/no)	-	90/235
**Sweating** (yes/no)	-	52/273
**Hemoglobin** (g/dL, median [IQR])	-	13.7 [12.6–14.6]

### Association of the genotypes and alleles of polymorphisms in *TLR5 R392StopCodon* and *TLR9 -1486C/T* with *P*. *vivax* malaria

Of all the SNPs studied, only *TLR9 -1486C/T* slightly deviated from the HWE in *Pv*-malaria cases (*p* = 0.006). *TIRAP S180L* was null in the population studied. The genotypic and allelic frequencies for all the SNPs are shown in Table 2. The genotype distributions for *TLR5 R392StopCodon* and *TLR9 -1486C/T* were significantly different between the groups (*p* = 0.03 and *p* = 0.01, respectively). The genotypes C/T (*TLR5 R392StopCodon*) and T/T (*TLR9 -1486C/T*) appear to be risk factors for *Pv-*infection (*TLR5*: C/C vs. C/T OR = 2.1 [95% CI: 1.1–4.5, *p* = 0.031]; TLR9: C/C vs. T/T OR = 1.9, [95% CI: 1.2–3.2, *p* = 0.01]; *TLR9*: C/C vs. C/T+T/T OR = 1.6, [95% CI: 1.1–2.5, *p* = 0.024]). Similar trends were observed at the allele levels for both SNPs. Carriers of the T allele were at higher risk for developing *Pv-*malaria (TLR5 OR = 2.1, [95% CI: 1.0–4.3, *p* = 0.034]; *TLR9* OR = 1.3, [95% CI: 1.0–1.7, *p* = 0.02]).

**Table 2:** Genotype and allele frequencies of the TLRs and CD14 polymorphisms in patients with *Pv*-malaria and healthy controls.

Polymorphism, Genotype or Allele	Healthy Group	*Pv*-malaria Cases	OR (IC 95%)	p-value	
	(n = 274)	(n = 325)			
***TLR1 I602S* (rs5743618)**					
**T/T**	153 (56%)	188 (58%)	0.9 (0.7–1.3)	0.621	T/T vs. T/G+G/G
**T/G**	107 (39%)	118 (36%)	0.9 (0.6–1.3)	0.530	T/T vs. T/G
**G/G**	14 (5%)	19 (6%)	1.1 (0.5–2.3)	0.787	T/T vs. G/G
**T**	413 (75%)	494 (76%)	1.0 (0.8–1.3)	0.798	
**G**	135 (25)	156 (24%)			
***TLR4 A299G* (rs4986790)**					
**A/A**	260 (95%)	312 (96%)	0.8 (0.4–1.7)	0.514	A/A vs. A/G
**A/G**	14 (5%)	13 (4%)			
**G/G**	-	-			
**A**	534 (97%)	637 (98%)	1.3 (0.6–2.8)	0.519	
**G**	14 (3%)	13 (2%)			
***TLR4 T399I* (rs4986791)**					
**C/C**	261 (95%)	310 (95%)	1.0 (0.5–2.1)	0.940	C/C vs. C/T
**C/T**	13 (5%)	15 (5%)			
**T/T**	-	-			
**C**	535 (98%)	635 (95%)	1.0 (0.5–2.2)	0.941	
**T**	13 (2%)	15 (5%)			
***TLR5 R392StopCodon* (rs5744105)**					
**C/C**	263 (96%)	298 (92%)	***2*.*1 (1*.*1–4*.*5)***	***0*.*031***	CC vs. CT
**C/T**	11 (4%)	27 (8%)			
**T/T**	-	-			
**C**	537 (98%)	623 (96%)	***2*.*1*** ***(1*.*0–4*.*3)***	***0*.*034***	
**T**	11 (2%)	27 (4%)			
***TLR6 S249P* (rs5743810)**					
**C/C**	4 (1%)	13 (4%)	1.0 (0.7–1.5)	0.889	C/C+C/T vs. T/T
**C/T**	73 (27%)	80 (25%)	0.3 (0.1–1.1)	0.057	C/C vs. C/T
**T/T**	197 (72%)	232 (71%)	0.4 (0.1–1.1)	0.068	C/C vs. T/T
**C**	81 15%)	106 (16%)	0.9 (0.7–1.2)	0.468	
**T**	467 (85%)	544 (84%)			
***TLR9 -1237C/T* (rs187084)**					
**T/T**	192 (70%)	222 (68%)	1.1 (0.8–1.5)	0.641	C/C vs. C/T+T/T
**C/T**	76 (28%)	93 (29%)	1.1 (0.7–1.5)	0.757	C/C vs. C/T
**C/C**	6 (2%)	10 (3%)	1.4 (0.5–4.0)	0.484	C/C vs. T/T
**T**	460 (84%)	537 (83%)	1.1 (0.8–1.5)	0.540	
**C**	88 (16%)	113 (17%)			
***TLR9 -1486C/T* (rs5743836)**					
**C/C**	56 (20%)	44 (14%)	***1*.*9 (1*.*2–3*.*2)***	***0*.*010***	C/C vs. T/T
**C/T**	153 (56%)	183 (56%)	1.5 (1.0–2.4)	0.065	C/C vs. C/T
**T/T**	65 (24%)	98 (30%)	***1*.*6 (1*.*1–2*.*5)***	***0*.*024***	C/C vs. C/T+T/T
**C**	265 (48%)	271 (42%)	***1*.*3*** ***(1*.*1–1*.*6)***	***0*.*020***	
**T**	283 (52%)	379 (58%)			
***CD14–159* (rs2569191)**					
**C/C**	91 (33%)	99 (31%)	1.2 (0.9–1.8)	0.244	C/C vs. C/T
**C/T**	118 (43%)	160 (49%)	1.2 (0.8–1.8)	0.313	C/C+C/T vs. T/T
**T/T**	65 (24%)	66 (20%)	1.3 (0.9–2.0)	0.173	C/T vs. T/T
**C**	300 (55%)	358 (55%)	1.0 (0.8–1.3)	0.908	
**T**	248 (45%)	292 (45%)			

### The *TLR9 -1237C/C* genotype is associated with increased parasitemia in *Pv*-malaria

Table 3 summarizes the univariable and multivariable linear regression analyses for parasitemia association with the different variables. Fever (COEF = 7599.46, 95% CI = 3063.80–12135.12, *p* = 0.001) and the C/C genotype of *TLR9 -1237C/T* (COEF = 17006.63, 95% CI = 3472.83–30540.44, *p* = 0.014) were independently associated with increased parasitemia.

**Table 3:** Association of parasitemia with *TLR* and *CD14* polymorphisms in *Pv*-malaria patients.

Variables	Parasitemia (number of copies / μL)			
	Crude COEF^a^ (IC 95%)	p-value	Adjusted COEF^b^ (IC 95%)	p-value
Age	-134.9 (-240.4 to -29.4)	***0*.*012***	-80.0 (-185.7 to 25.6)	0.137
Gender (Male)	1	-	1	-
Female	-1305.8 (-6,131.9 to 3,520.1)	0.594	-	-
Fever (No)	1	-	1	-
Yes	8630.2 (4,114.1 to 13,146.2)	***<0*.*0001***	7599.5 (3,063.8 to 12,135.1)	***0*.*001***
Hemoglobin	-661.7 (-2,160.9 to 837.4)	0.385	-	-
*TLR1 602* (G/G)	1	-	1	-
(T/G)	1872.6 (-8,143.7 to 11,888.9)	0.713	-	-
(T/T)	5111.41 (-4,702.3 to 14,925.1)	0.306	-	-
*TLR4 299* (A/A)	1	-	1	-
(A/G)	-4766.5 (-17,680.3 to 8,147.3)	0.468	-	-
*TLR4 399* (C/C)	1	-	1	-
(C/T)	-3050.6 (-14,503.7 to 8,402.4)	0.600	-	-
*TLR5 392* (C/C)	1	-	1	-
(C/T)	5609.1 (-3,372.2 to 14,590.5)	0.220	-	-
*TLR6 249* (C/C)	1	-	1	-
(C/T)	3510.9 (-8,571.6 to 15,593.3)	0.567	-	-
(T/T)	5610.6 (-5,934.8 to 17,156.1)	0.339	-	-
*TLR9–1237* (T/T)	1	-	1	-
(C/T)	2006.9 (-3,259.7 to 7,273.4)	0.453	-	-
(C/C)	21633.9 (7,922.5 to 35,345.3)	***0*.*002***	17006.6 (3,472.8 to 30,540.4)	***0*.*014***
*TLR9–1486* (C/C)	1	-	1	-
(C/T)	-155.6 (-7,297.8 to 6,986.6)	0.966	-	-
(T/T)	-3133.6 (-10,963.1 to 4,695.9)	0.431	-	-
*CD14–159* (C/C)	1	-	1	-
(C/T)	-1374.1 (-6,668.6 to 3,920.4)	0.609	-	-
(T/T)	2708.8 (-4,073.2 to 9,490.9)	0.432	-	-

^a^Univariable regression linear model.

^b^Multivariable regression linear model.

### Linkage disequilibrium of the *TLR* polymorphisms

Linkage disequilibrium (LD) between polymorphisms in receptors *TLR1* and *TLR6* (*I602S* vs. *S249P*), *TLR4* (*A299G* vs. *T399I*), and *TLR9* (*-1237C/T* vs. *-1486C/T*) was very low, as calculated by Haploview 4.2 software. The R^2^ and D' of the LD were 0.38 and 0.83 for *TLR1* and *TLR6*, 0.18 and 0.44 for *TLR4*, 0.006 and 0.20 for *TLR9*.

## Discussion

*Pv*-malaria is still considered a neglected disease despite various efforts directed to its control and elimination [44–46]. *P*. *vivax* presents biological complexity due to the interplay between environmental factors, parasite load, and the immunological status and genetic background of the human host [17,47]. The hypnozoite stage in the liver can cause clinical episodes of relapse with lower parasite load. These are often associated with mild or asymptomatic clinical display and contribute to the transmission of the disease [48,49]. The molecular mechanisms influencing these characteristics remain poorly understood. Knowledge of the host genetic factors in malaria may contribute to the elucidation of the molecular mechanisms involved in the development of the disease, as not all individuals exposed to the parasites develop symptoms. The identification of genes involved in susceptibility or resistance to infection by *P*. *vivax* is important to understand the pathogenesis of the disease and may contribute to the designing of control and elimination tools, as well as the development of an effective vaccine.

Innate immunity plays a key role in infectious processes. The discovery of pattern recognition receptors (PRRs) such as TLRs, Nod-like receptors (NLRs), RIG-I-like receptors (RLRs), and scavenger receptors has contributed to the understanding of infectious disease [50,51]. TLRs are key mediators in the response to malaria, playing an important role in the bridge between innate and adaptive immunity, mainly by activation of transcription factor NF-κB and inducing production of proinflammatory cytokines [52]. SNPs in TLRs and adapter molecules that influence the inflammatory process and proinflammatory cytokine production may be associated with susceptibility to infections [34,53,54]. Changes in the production of these cytokines may influence the control of parasitemia and clinical manifestations of the disease, since a fine balance in the inflammatory process is essential for parasite clearance [15,16,55,31]. The data presented in this study suggest an association of polymorphisms *TLR5* and *TLR9* with *Pv*-malaria. Furthermore, the SNP *TLR9* -*1237C/T* was associated with increased parasitemia.

Allelic variants of *TLR1 I602S* and *TLR6 S249P* showed no association with *Pv-*malaria. An association of polymorphisms in *TLR1* and *TLR6* with mild malaria in patients infected with different *Plasmodium* species was recently demonstrated. Variants in *TLR1* may predispose patients with *Pf-*malaria complications and increased parasitemia [24,25]. In addition, SNPs *I602S* (*TLR1*) and *S249P* (*TLR6*) were associated with severe malaria in Indian patients, showing the genetic contribution of these variants to the onset of cerebral malaria [56]. TLR-1 and -6 form heterodimers with TLR-2 and recognize the GPI anchor of the parasite. Mutations in these receptors can impair recognition and subsequent elimination of *Plasmodium* [12,24].

SNPs in *TLR4* were not associated with *Pv*-malaria in our study. These polymorphisms have been associated with severe manifestations of malaria in children and mild symptoms in pregnant African women infected with *P*. *falciparum* [26,27]. Furthermore, *T399I* and *A299G* in *TLR4* were associated with increased parasitemia in Indian patients with *Pf*-malaria, indicating that this receptor is important in inducing immune response to malaria [57]. In addition, these SNPs appeared to modulate the susceptibility to severe anemia and malaria in Nigerian children [58,59]. Moreover, there was no correlation between these polymorphisms and complications of malaria in adult patients and in other parasitic diseases, such as chronic Chagas disease [28,60,61], and this study corroborates this lack of association. The *TLR4/CD14* complex is responsible for cytokine production via NF-κB, and the SNP *CD14*–*159* in the promoter region has been associated with susceptibility to tuberculosis, and was shown to influence the production of IFN-γ [62–64]. Our data does not show any association of this SNP with *Pv*-malaria or parasitemia.

Flagellin (FliC), present in bacteria and parasites, is a ligand of TLR-5. Activation of TLR-5 leads to the production of proinflammatory cytokines such as IL-6 [65]. To our knowledge, this is the first report of an association of the SNP *R392StopCodon* in *TLR5* with susceptibility to *Pv*-malaria. TLR-5 was shown to be a promising alternative to enhance the immunogenicity of the proteins to specific *P*. *vivax*, such as the 19 kDa C-terminal fragment of merozoite surface protein 1 (MSP1_19_) after combination with *Salmonella enterica* serovar Typhimurium FliC [66,67]. Thus, we suggest that individuals with this mutation may not react favorably to this vaccine design. Alternative vaccines for carriers of this mutation must be sought.

The association of the *-1486C/T* variant present in the promoter region of *TLR9* with *Pv*-malaria in this study corroborated other observations of association of this SNP with susceptibility to symptomatic and placental malaria caused by *P*. *falciparum* [24,27]. Interestingly, this SNP has been shown to correlate with high *Plasmodium* parasitemia and low production of IFN-γ and TNF-α [24,68,69]. We show the genotype C/C of the SNP *TLR9* -*1237C/T* was associated with high parasite load. Together, these data confirm recent studies with *Pf*-malaria and suggest that TLR9 may play a key role in controlling parasitemia [24,69]. *TLR9*-depleted mice showed loss in control of parasitic infections compared to wild type mice [23,70]. SNPs in *TLR9* appear to predispose Indian individuals to severe malaria [71] and these findings were confirmed in a meta-analysis study with 665 severe malaria patients and 1,187 uncomplicated malaria individuals from India and Africa, with an association of variants *-1486C/T* and *-1237C/T* of *TLR9* with severe malaria [72].

This study has some limitations. Although the levels of associations with *Pv*-malaria and parasitemia are high, the study population is small. It needs validation with a larger sample size to confirm the importance of *TLR9* and *TLR5* in *Pv*-malaria. The small sample size does not allow intra-comparison of the genotypes and alleles studied with clinical manifestations of *Pv*-malaria, including in asymptomatic, mild, and severe malaria.

To our knowledge, this is the first study to show that variants in the TLR pathway may be involved in the pathogenesis of *P*. *vivax* malaria. TLRs are key mediators in response to malaria as they trigger the expression of proinflammatory cytokines to inhibit parasite growth. *TLR5 R392StopCodon* and *TLR9 -1486C/T* may predispose individuals to *P*. *vivax* malaria, while *TLR9 -1237C/T* was associated with *Pv-*malaria with high parasitemia. However, additional studies should be conducted in other endemic areas to confirm the role of host genetics in infection and pathogenesis of *Pv*-malaria.

## Supporting information

S1 Table Description of assay, primer/probe sequences, qPCR protocol, qPCR efficiency and detection limit for quantification of *P*. *vivax* DNA. (DOCX)

S2 Table Description of polymorphisms, primer sequences, PCR protocols, restriction enzymes, and fragments generated during the SNP identification study. (DOCX)
