# Supplementary material for: Association of TLR variants with susceptibility to Plasmodium vivax malaria and parasitemia in the Amazon region of Brazil
Source: PLoS One. 2017 Aug 29;12(8):e0183840. doi: 10.1371/journal.pone.0183840 (PMC5574562; doi:10.1371/journal.pone.0183840)
Supplement: S1 Table — (DOCX) [file pone.0183840.s001.docx]

**S1 Table: Description of assay, primer/probe sequences, qPCR protocol, qPCR efficiency and detection limit for quantification of *P. vivax* DNA.**

| **Assay** | **Primers/Probe** | **qPCR Protocol** | **qPCR Efficiency** | **Assay detection limit** |
| --- | --- | --- | --- | --- |
| *P. vivax-specific*  *(18S rRNA gene)* | F^*^: 5’-GCTTTGTAATTGGAATGATGGGAAT-3’  R^#^: 5’-ATGCGCACAAAGTCGATACGAAG-3’  P^¥^: VIC-AGCAACGCTTCTAGCTTA-MGB-NFQ | 50°C for 2min, 95°C for 10 min,  45x (95°C for 15s, 58°C for 1 min) | 92.2% | 1 copy/uL |

^*^F: Forward; ^#^R: Reverse; ^¥^P: Probe.
