# Supplementary material for: Association of TLR variants with susceptibility to Plasmodium vivax malaria and parasitemia in the Amazon region of Brazil
Source: PLoS One. 2017 Aug 29;12(8):e0183840. doi: 10.1371/journal.pone.0183840 (PMC5574562; doi:10.1371/journal.pone.0183840)
Supplement: S2 Table — (DOCX) [file pone.0183840.s002.docx]

**S2 Table: Description of polymorphisms, primer sequences, PCR protocols, restriction enzymes, and fragments generated during the SNP identification study.**

| **Polymorphisms** | **Primers** | **PCR Protocols** | **Restriction enzymes** | **Alleles and fragments (bp^¤^)** |
| --- | --- | --- | --- | --- |
| *TLR1 I602S*  *(rs*5743618)* | F^#^: 5’-GGAAAGTTATAGAGGAACCCT-3’  R^¥^: 5’-CTTCACCCAGAAAGAATCGTGCC-3’ | 95°C for 5min, 40x (95°C for 30s, 55°C for 30s, 72°C for 30s), 72°C for 7min | *AluI* | T: 280  G: 151+129 |
| *TLR4 A299G*  *(rs4986790)* | F: 5’-ATACTTAGACTACTACCTCCATG-3’  R: 5’-AAACTCAAGGCTTGGTAGATC-3’ | 95°C for 5min, 35x (95°C for 30s, 56°C for 30s, 72°C for 30s), 72°C for 7min | *NcoI* | A: 259  G: 239+20 |
| *TLR4 T399I*  *(rs4986791)* | F: 5’-GCTGTTTTCAAAGTGATTTTGGGAGAA-3’  R: 5’-CACTCATTTGTTTCAAATTGGAATG-3’ | 95°C for 5min, 35x (95°C for 30s, 60°C for 30s, 72°C for 45s), 72°C for 5min | *Hinf-I* | C: 147  T: 96+51 |
| *TLR5 R392StopCodon (rs5744105)* | F: 5’-GGTAGCCTACATTGATTTGC-3’  R: 5’- GAGAATCTGGAGATGAGGTACCCG-3’ | 95°C for 5min, 40x (95°C for 30s, 62°C for 30s, 72°C for 30s), 72°C for 7min | *DdeI* | C: 277pb  T: 186+91 |
| *TLR-6 S249P (rs5743810)* | F: 5’-GCATTTCCAAGTCGTTTCTATGT-3’  R: 5’-GCAAAAACCCTTCACCTTGTT-3’ | 95°C for 5min, 40x (95°C for 30s, 63°C for 30s, 72°C for 30s), 72°C for 7min | *AvaII* | C: 210  T: 160+50 |
| *TLR9 -1237C/T (rs187084)* | F: 5’-CTGCTTGCAGTTGACTGTGT-3’  R: 5’-ATGGGAGCAGAGACATAATGGA-3’ | 95°C for 5min, 40x (95°C for 30s, 59°C for 30s, 72°C for 45s), 72°C for 7min | *BstNI* | C: 108+27  T: 60+48+27 |
| *TLR9 -1486C/T (rs5743836)* | F: 5’-TATCGTCTTATTCCCCTGCTGGAATGT-3’  R: 5’-TGCCCAGAGCTGACTGCTGG-3’ | 95°C for 5min, 40x (95°C for 30s, 59°C for 30s, 72°C for 30s), 72°C for 7min | *AflII* | C: 145  T: 111+34 |
| *TIRAP S180L*  *(rs8177374)* | F: 5’-TGCTCATCACGCCGGGCTTCCTT-3’  R: 5’-TAGGCAGCTCTGCTGAGGTCC-3’ | 95°C for 5min, 35x (95°C for 30s, 62°C for 30s, 72°C for 30s), 72°C for 7min | *Hpy188I* | C: 106+21  T: 127 |
| *CD14 -159*  *(rs2569191)* | F: 5’-GTGCCAACAGATGAGGTTCAC-3’  R: 5’-GCCTCTGACAGTTTATGTAATC-3’ | 95°C for 5min, 35x (95°C for 30s, 65°C for 30s, 72°C for 30s), 72°C for 7min | *AvaII* | C: 497  T: 353+144 |

*rs: Reference sequence; ^#^F: Forward; ^¥^R: Reverse; ^¤^bp: Base pairs.
